# Safety and efficacy of Evermine50 Everolimus-eluting coronary stent system in patients with native coronary artery lesions: Three-year outcomes from a single-center

**DOI:** 10.34172/jcvtr.025.33123

**Published:** 2025-03-18

**Authors:** Suresh V Patted

**Affiliations:** Department of Cardiology, KLE Academy of Higher Education & Research, Belagavi, Karnataka, India

**Keywords:** Native coronary artery lesions, Evermine50 Everolimus-eluting stent, Major adverse cardiac events, Stent thrombosis, Ultrathin strut

## Abstract

**Introduction:**

The Evermine50^TM^ (Meril Life Sciences Pvt. Ltd., India) is the world’s thinnest strut (50 µm) featuring a biodegradable polymer-based Everolimus-eluting stent (EES) system. We present the 3-year safety and performance outcomes of Evermine50 EES.

**Methods:**

This was a prospective, post-marketing, single-center study of patients with native coronary artery lesions (CAL) in real-world settings. Patients with symptomatic ischemic heart disease due to *de novo* and in-stent restenotic lesions (lengths<44mm) in native coronary arteries with reference vessel diameters of 2.0 - 4.5 mm. and eligible for stenting procedure with percutaneous transluminal coronary angioplasty were included.

**Results:**

A total of 251 patients (mean age: 58.20 years) were enrolled, of which 48.2% had ST-elevation myocardial infarction and 31.5% had silent ischemia. The mean lesion length was 21.81±8.14 mm, and 70.3% of patients had pre-procedure Thrombolysis in Myocardial Infarction (TIMI) flow grade III. The average and stent length was recorded as 23.50±12.21 mm. In 98% of patients, post-procedural TIMI-III flow grade was achieved. The cumulative rate of major adverse cardiac events defined as composite of cardiac death, target vessel myocardial infarction, and clinically-driven target lesion revascularization (CD-TLR) at 1, 2, and 3 years were 1.59%, 3.58%, and 3.58%, respectively. The cumulative rates of CD-TLR remained constant at 0.79% from 1 to 3 years. There were no cases of stent thrombosis until 3 years.

**Conclusion:**

This study demonstrated favorable safety and performance of the ultrathin Evermine50 EES at 36 months in patients with native CAL.

## Introduction

 Percutaneous coronary intervention (PCI) is the primary therapeutic approach for managing coronary artery disease (CAD). It is estimated that more than two million PCI procedures are performed worldwide every year. Nevertheless, there are certain long-term complications associated with the procedure. These include restenosis, stent thrombosis (ST), and bleeding complications, among others. Therefore, there has been ongoing research and innovation to improve PCI outcomes.^1^ The field has been continually evolving with developing and enhancing technologies and devices.^2^

 The first transition was from bare metal stents (BMSs) to drug-eluting stents (DES). Drug-eluting stents (DESs) were developed to reduce neointimal hyperplasia and restenosis after PCI which was common with BMS. However, first-generation DESs were associated with an increased risk of ST than BMS.^3^ First-generation DES reduced neointimal hyperplasia (NIH) compared to BMS but were associated with impaired vascular healing, resulting in incomplete endothelial coverage. The presence of permanent polymers may have delayed healing due to hypersensitivity reactions, and blood exposure to thrombogenic stent struts increases the risk of stent thrombosis.^4^

 Subsequently, further advancements in technology led to the development of second-generation DESs. Traditionally, BMSs were manufactured from stainless steel. In contrast, the latest generation of DESs utilizes metallic platforms made from cobalt-chromium (CoCr) or platinum-chromium (PtCr) alloys.^2^ Compared to the first-generation DES, second-generation DES have thinner struts and more biocompatible polymers, which reduce vascular injury and inflammation.^5^ This is because thinner struts have a smaller surface area, which reduces the time taken for endothelialization, thereby reducing the risk of restenosis. Furthermore, thinner struts cause less vessel injury and local inflammation, which reduces the risk of NIH and ST.^4^ Reducing strut thickness from 130–140 µm in the first-generation DESs to 60–80 µm in the second-generation also promotes faster endothelial coverage following implantation.^2^ New stents also use a lower drug load to facilitate the endothelization process.^2^ Contemporary second-generation DESs have considerably improved PCI outcomes; studies have shown a lower risk of restenosis, ST, and myocardial infarction (MI) and improved survival.^6^ Despite advancements, conventional second-generation thin-strut DES still face risks of adverse clinical events even after the first year of implantation.^7^ Thus, clinical outcomes with contemporary second-generation DES have plateaued with no significant improvements in the last decade.^6^

 In recent times, stents with struts have been introduced. These have the potential to reduce vascular injury further and accelerate endothelialization.^6^ Ultrathin DESs are characterized by a strut thickness measuring below 70 µm.^2^ Earlier meta-analyses have indicated that ultrathin-strut DESs significantly lower the one-year incidence of target lesion failure (TLF) compared to conventional second-generation thin-strut DES.^6,7,8^ Moreover, ultrathin stents are more flexible, trackable, and easily deliverable across complex lesions, especially in smaller vessels (2.25-2.5 mm).^9^

 The Evermine50^TM^ (Meril Life Sciences Pvt. Ltd., India) is the world’s thinnest ultrathin strut (50 µm) with biodegradable polymer-based Everolimus-eluting stent (EES) system. The 6-,12-, and 24-month safety and efficacy outcomes with this stent have been previously reported.^10-12^ We now report the 36-month outcomes with Evermine50 EES in patients with native coronary artery lesions in real-world settings.

## Materials and Methods

 This was a prospective, post-marketing, single-center, study to evaluate the safety and performance of the Evermine50 EES (50μm) in the treatment of patients with *de novo* native coronary artery lesions in real-world settings. Patients presenting with symptomatic ischemic heart disease attributed to *de novo* and in-stent restenosis lesions (< 44 mm length) within native coronary arteries, having a reference vessel diameter between 2.0 mm and 4.5 mm, were considered for inclusion if they qualified for percutaneous transluminal coronary angioplasty (PTCA) and stenting interventions. The 36-month follow-up is presented in the current investigation.

 This study was conducted according to the International Council for Harmonization of Technical Requirements for Pharmaceuticals for Human Use (ICH) standards for clinical research, including ICH E6 [Good Clinical Practice (GCP)] and ICH E3 (Study Reporting); International Organization for Standards (ISO) 14155 standards for Conduct of the study. The local regulations were followed in data management quality assurance. It was registered on the clinical trials registry (CTRI number: CTRI/2017/03/008173). The research complied with the Declaration of Helsinki and got approval from the Institutional Review Board/Institutional Ethics Committee.

###  Study Device

 The study device has been previously described in detail.^10^ The device used was Evermine50 EES (Meril Life Sciences Pvt. Ltd., India), a stent with the thinnest (50 µm) strut. It is built on a cobalt-chromium platform and is coated with biodegradable polymers: Poly-lactic-co-glycolic acid (PLGA) and Poly-L-lactic acid (PLLA). The hybrid cell design used by Evermine50 EES is distinctive and combines an unique combination of open cells in the middle and closed cells at the edges. It elutes everolimus (1.25 µg/mm^2^ of stent area) as an anti-proliferative drug.^10^

###  Study Endpoints

 The safety endpoint was a composite of major adverse cardiac events (MACE) and ST at 1 month ( ± 14 days), 6 months ( ± 28days), 12 months ( ± 28 days), 24 months ( ± 28 days) and 36 months ( ± 28 days), respectively. The MACE and ST were defined as per Academic Research Consortium (ARC)-2 guidelines. MACE was defined as the composite of cardiac death, target vessel MI, and clinically driven target lesion revascularization (CD-TLR). Cardiac death was defined as any death resulting from an acute MI, sudden cardiac death, death due to heart failure, or death due to stroke. CD-TLR refers to the revascularization procedure conducted in patients who exhibit clinical symptoms, such as unstable angina. ST was defined as the presence of a thrombus originating in the stent or in the segment 5 mm proximal or distal to the stent and the presence of at least one of the following criteria within a 48-hour time window: a) Acute onset of ischemic symptoms at rest b) New ischemic electrocardiographic (ECG) changes suggestive of acute ischemia. c) Typical rise and fall in cardiac biomarkers.

 The performance endpoint composed of ischemic-driven target lesion revascularization (ID-TLR) and ischemia-driven target vessel revascularization (ID-TVR) at 1 month ( ± 14 days), 6 months ( ± 28days), 12 months ( ± 28 days), 24 months ( ± 28 days) and 36 months ( ± 28 days) respectively. The performance endpoint also comprises procedural and device success. ID-TLR was defined as any repeat PCI of the target lesion, bypass surgery of the target vessel due to restenosis, or any other complication in the target lesion. ID-TVR was defined as any repeat PCI or surgical bypass of any segment of the target vessel. Procedural success was established as attaining technical success without MACE occurring within 24 hours following the index procedure. Device success is defined as angiographic evidence of less than 30% final residual stenosis of the target lesion using only the assigned device. Silent ischemia was typically defined as objective evidence of myocardial ischemia in patients without symptoms related to that ischemia. Silent ischemia may be detected in patients who have no symptoms during an exercise or pharmaceutical stress test but with transient ST-segment changes, perfusion defects, or reversible regional wall motion abnormalities.^13^

###  Eligibility Criteria

*Inclusion criteria:* Patients aged > 18 years, and willing to participate in the study. The subject’s coronary artery must be suitable for implantation of Evermine50 EES, and implantation of only Evermine50 EES stent(s) during the index procedure was considered. The female subjects of childbearing potential with negative pregnancy test results and not breast-feeding at the time of screening should not plan for conception for up to 2 years from the date of index procedure. Subjects who agreed to not participate in any other investigational or invasive clinical study for 2 years from the date of the index procedure were considered.

*Exclusion criteria:* Individuals unable to provide informed consent, as well as those with documented allergies or hypersensitivity to aspirin, heparin, Everolimus, polymer lactide, cobalt-chromium alloys, and glycolide antiplatelet medications (including clopidogrel and prasugrel), are not eligible for participation. Females with known pregnancy or lactating at the time of screening were excluded from the study.

###  Procedure and post-PCI therapy

 Subjects were implanted with Evermine50 EES for *de novo* coronary artery lesions as per the standard guidelines and practices. They were maintained on a standard anti-coagulation regimen during the post-operative and follow-up periods. The clinical follow-up was conducted at 1 month ( ± 14 days), and subsequent telephonic/clinical follow-ups were conducted at 6 months ( ± 28 days), 12 months ( ± 28 days), 24 months ( ± 28 days) and 36 months ( ± 28 days).

 Dual antiplatelet therapy (DAPT), consisting of ticagrelor or clopidogrel along with aspirin, or single antiplatelet therapy (SAPT) when required, was prescribed for a minimum duration of one year.

###  Sample size calculation

 A previous study reported that MACE as a major endpoint occurred in 6.8% of patients, including cardiac death (1.7%), MI (1.5%), and ID-TLR (4.2%).^14^ Assuming a similar proportion of clinical outcomes pattern among the study population, a sample size of approximately 250 patients with a two-sided 95% confidence interval and 0.035 exact half widths of the confidence interval (Clopper-Pearson) and with an assumed 10% dropout rate will be considered for the study analysis.

###  Statistical Analysis

 All subjects lost to follow-up after 1-month were included for efficacy evaluation based on the Last Observation Carried Forward (LOCF). All dropouts and those lost to follow-up subjects were excluded from the safety analysis.

 The baseline and demographic data were presented as descriptive statistics. The mean and standard deviation were used to present continuous variables such as age and body mass index. The categorical variables, including gender, risk factors, and cardiac status, were reported as frequencies and percentages. The percentages were calculated based on the number of patients for whom data were available. All analyses were conducted using SPSS version 15.

## Results

**Figure 1:**
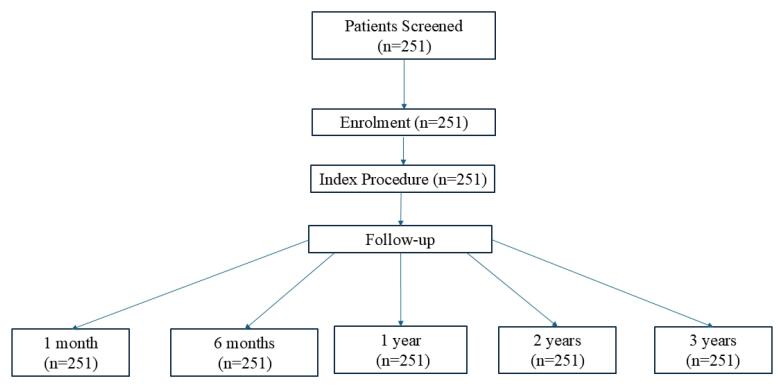
Study flowchart

 The summary of the patient disposition flow diagram is shown in Figure 1. A total of 251 patients were enrolled. The mean age was 58.20 ± 9.92 years, and 76.9% were male. The baseline characteristics of patients are demonstrated in Table 1. Of the total cohort, 83.7% had angina. The proportion of patients with diabetes and hypertension was 48.6% and 45.4%, respectively.

**Table 1:** Baseline and Demographic Characteristics

**Variables**	**n=251**
Age, years, mean ± SD	58.20 ± 9.92
Male, n (%)	193 (76.9)
Female, n (%)	58 (23.11)
Body mass index, kg/m^2^, mean ± SD	25.02 ± 3.37
Systolic blood pressure, mmHg, mean ± SD	126.23 ± 17.97
Diastolic blood pressure, mmHg, mean ± SD	78.72 ± 7.74
Diabetes mellitus	122 (48.6)
Hypertension	114 (45.4)
Smoking	38 (15.1)
Dyslipidemia	15 (5.9)
Alcoholic	29 (11.6)
COPD	2 (0.8)
Ischemic heart disease	207 (82.5)
Family history of CAD	28 (11.2)
PCI/ MI/CABG	16 (6.4)
Angina	209 (83.7)
Stroke	4 (1.6)
**Cardiac Status**	
Unstable angina	26 (10.4)
Stable angina	2 (0.8)
STEMI	121 (48.2)
NSTEMI	23 (9.2)
Asymptomatic/Silent ischemia	79 (31.5)
LVEF, % (mean ± SD)	50.89 ± 8.24

CABG: Coronary artery bypass graft; CAD: coronary artery disease; COPD: Chronic obstructive pulmonary disease; LVEF: Left ventricular ejection fraction; MI: Myocardial infarction; NSTEMI: Non-ST-elevation myocardial infarction; PCI: Percutaneous coronary intervention; STEMI: ST-elevation myocardial infarction

 The mean lesion length was 21.81 ± 8.14 mm and 70.3% of patients had a pre-procedure Thrombolysis in Myocardial Infarction (TIMI) flow grade III. Most patients had American Heart Association (AHA) lesion class A (31.5%) or B1 (46.6%). The lesion characteristics are shown in Table 2.

**Table 2:** Lesion characteristics

**Variables, n (%) **	**n=251 Patients**
Diseased Vessels	
Single vessel	124 (49.4)
Double vessel	88 (35.1)
Triple vessel	39 (15.54)
Average lesion length, mm, Mean ± SD	21.81 ± 8.14
Treated Lesion location, n (%)	
Right coronary artery	107 (31.2)
Left anterior descending artery	173 (50.4)
Left circumflex artery	58 (16.9)
Ramus	5 (1.5)
TIMI flow pre-procedure, n (%)	
0	38 (11.1)
1	29 (8.5)
2	35 (10.2)
3	241 (70.3)
Stenosis type, n (%)	
de novo	337 (98.2)
In-stent	3 (0.9)
Bifurcation	3 (0.9)
AHA Lesion class, n (%)	
A	108 (31.5)
B1	160 (46.6)
B2	21 (6.1)
C	54 (15.7)
Lesion type, n (%)	
Calcified	5 (1.5)
Diffused	9 (2.6)
Chronic total occlusions	6 (1.7)
Thrombus	32 (9.3)
Long	102 (29.7)
Other	189 (55.1)
Discrete	187 (54.5)
Thrombus, Spiral discrete	1 (0.3)
Non flow limiting lesion	1 (0.3)

AHA: American Heart Association; TIMI: Thrombolysis in Myocardial Infarction

 A total of 343 lesions were treated. The commonest stent lengths used were 16, 19, 24, and 29 mm; the average stent length was 23.50 ± 12.21 mm. The average stent diameter was 2.83 ± 0.23 mm. The device and procedure success rates were 100%. The procedural characteristics are summarized in Table 3. In line with current guidelines for ACS management, dual antiplatelet therapy (DAPT) was administered to patients in this study. Among the cohort, 89.6% received ticagrelor, 6.8% received clopidogrel, 3.2 received prasugrel, and the remaining patients were managed with single antiplatelet therapy (SAPT), primarily aspirin, based on the clinical judgment of the treating physicians.

**Table 3:** Procedural characteristics

**Characteristic**	**343 lesions/251 patients**
Total number of lesions treated with study stent	343
Study stents per patient (%)	1.37
Total number of study stents used	343
Access Site, n (%)	
Femoral right	249 (99.20)
Femoral left	2 (0.80)
Stent length, mm, n (%)	
8	1 (0.29)
13	22 (6.41)
16	62 (18.07)
19	78 (22.74)
24	54 (15.74)
29	45 (13.12)
32	34 (9.91)
37	19 (5.54)
40	28 (8.16)
Stent diameter, mm, n (%)	
2.0	1 (0.29)
2.25	2 (0.58)
2.5	17 (4.96)
2.75	55 (16.03)
3.0	104 (30.32)
3.5	145 (42.10)
4.0	18 (5.25)
4.5	1 (0.29)
Contrast Media Used	
Ionic, n (%)	22 (8.76)
Non-ionic, n (%)	229 (91.24)
Average stent length, mm, Mean ± SD	23.50 ± 12.21
Average stent diameter, mm, Mean ± SD	2.83 ± 0.23
TIMI flow post-procedure, n (%)	
0	4 (1.2)
1	0
2	3 (0.9)
3	336 (98)
Pre-dilatation, n (%)	262 (76.4)
Post-dilatation, n (%)	196 (57.1)
Procedure Success, n (%)	251 (100)
Device Success, n (%)	251 (100)

TIMI: Thrombolysis in Myocardial Infarction

 The cumulative 36-month outcomes are shown in Table 4. The rate of MACE at 6-,12-,24-, and 36-months were 0.8%, 1.59%, 3.58%, and 3.58%, respectively. There were no cases of ST until 36 months. The rates of CD-TLR and ID-TVR were very low and remained constant at 0.79% from 12 to 36 months.

**Table 4:** Cumulative clinical events until 36 months follow-up

**Clinical Events, n (%)**	**In-hospital**	**1 month**	**6 months**	**12 months**	**24 months**	**36 months**
	**n=251**	**n=251**	**n=251**	**n=251**	**n=251**	**n=251**
All-cause death	0	0	2 (0.8)	6 (2.39)	12 (4.78)	17 (6.77)
Cardiac death*	0	0	1 (0.4)	2 (0.79)	7 (2.78)	7 (2.78)
Non-cardiac death	0	0	1 (0.4)	4 (1.59)	5 (1.99)	10 (3.88)
Myocardial infarction*	0	0	1 (0.4)	2 (0.79)	7 (2.78)	7 (2.78)
CD-TLR	0	0	1 (0.4)	2 (0.79)	2 (0.79)	2 (0.79)
ID-TVR including TLR	0	0	1 (0.4)	2 (0.79)	2 (0.79)	2 (0.79)
Stent thrombosis	0	0	0	0	0	0
MACE	0	0	2 (0.8)	4 (1.59)	9 (3.58)	9 (3.58)

CD-TLR: Clinically-driven target lesion revascularization; ID-TVR: Ischemia-driven target vessel revascularization; MACE: Major adverse cardiac event; n: number of patients. *7 patients suffered from MI and cardiac death.

## Discussion

 Our study with the Evermine50 EES showed low MACE, CD-TLR, and cardiac death rates at 3 years. Moreover, there was no case of ST until the 3-year follow-up period, and the cumulative rates of CD-TLR and ID-TVR remained low and constant for 1 to 3 years. The rate of MACE also remained constant from 24 to 36 months. Although the study cohort was predominantly skewed toward acute cases, such as STEMI (48.2%), it also included a diverse range of lesion complexities, including long lesions (29.7%) and thrombotic lesions (9.3%). This diversity, despite the limited representation of highly complex cases like bifurcations and CTOs, provides valuable insights into the performance and versatility of the Evermine50 stent across different clinical and lesion profiles in real-world settings. Next, the predominance of femoral access in this study reflects the standard practice at the participating center, particularly for high-risk STEMI cases requiring larger catheters. While radial access is increasingly favored, femoral access remains crucial for complex cases and highlights the importance of operator expertise.^15^

 Another study reported the 1-year outcomes with Evermine50 EES in 711 patients in whom long stents (median stent per lesion 54 ± 14 mm) were used. The rates of cardiac deaths, ID-TVR, target vessel failure (TVF), and ST were 1.2%, 3.2%, 7.7%, and 0.8%, respectively.^9^ These data indicate the good safety and efficacy of Evermine50 EES. These outcomes could be due to the ultrathin strut (50 µm) of the Evermine50 EES, the world’s thinnest strut. Moreover, the unique design (thinnest strut with hybrid cell) makes it promptly trackable and deliverable in most conventional lesion types.^9^

 Other studies on the outcomes of ultrathin stents have been published. The BIOFLOW-IV trial compared the Orsiro ultrathin stent (Biotronik, Oswega, USA) with the Xience (Abbott Vascular, Santa Clara, USA) second-generation stent. The Orsiro stent has a 60 µm strut thickness; it is made of a cobalt-chromium alloy and elutes sirolimus. At 12 months, the rate of TVF (composite of cardiac death, target vessel MI, emergent coronary artery bypass graft, and CD-TVR) was observed to be 5.5% in the Orsiro cohort, while the Xience cohort showed a rate of 7.5%.^16^ The BIOFLOW-V trial also compared Orsiro with Xience. At 3 years, the TLF was 8.2% for the Orsiro stent and 13.6% for the Xience stent (p = 0.002), primarily due to lower target-vessel MI (5.0% vs. 9.2%; *P* = 0.003) and CD-TLR (3.2% vs. 6.7%; *P* = 0.006) in the Orsiro group. Additionally, the definite or probable late stent thrombosis rate was significantly lower in the Orsiro group at 0.1% compared to 1.2% for Xience (*P* = 0.018).^17^ Other Orsiro trials include the BIOSTEMI and SORT OUT VII trials.^18-19^ In the BIOSTEMI trial, the TLF rate was 5.1%, and TLR was 2.5% in the Orsiro group at 2 years. In the SORT OUT VII trial, TLF (composite of cardiac death, MI, and TLR) at 12 months was 3.8% in the Orsiro group, and definite ST was 0.4%, which increased to 1% at 3 years. Our outcomes are not inferior to those of studies that used Orsiro SES in patient populations, like those in our study. The DESSOLVE III trial compared the MiStent ultrathin stent (Micell Technologies, Durham, NC) with the second-generation Xience DES. MiStent has a 64 µm strut thickness SES and is made of a cobalt–chromium alloy. In the DESSOLVE III study, the device-oriented composite endpoint of cardiac death, target-vessel MI, or clinically indicated TLR at 12 months was 5.8% in the MiStent group and 6.5% in the Xience group. The rate of ST was 0.7%. At 2 years and 3 years, the rate of the device-oriented composite endpoint in the MiStent group was 8.7% and 10.5%, respectively. The additional 3-year outcomes comprised of cardiac death (3.9%), target-vessel MI (3.2%), clinically indicated TLR (5.2%), and ST (1.2%).^20^ Our outcomes are much superior to those of the MiStent ultrathin stent. The TALENT trial compared the outcomes of Supraflex ultrathin stent, a 60 μm SES, made from L605 cobalt–chromium alloy with the second-generation Xience DES. The composite endpoint of cardiac death, target-vessel MI, or clinically indicated TLR at 12-month follow-up was seen in 4.9% of subjects in the Supraflex stent group (Sahajanand Medical Technologies, Surat, India) and 5.3% of the Xience stent group. This increased to 6.9% and 7.9%, respectively, at 2 years. The cardiac death rates in the Supraflex group at 1- and 2 years were 1% and 1.3% and the ST rates were 0.8% and 1.1%, respectively.^21^ Our outcomes were superior to those of the Supraflex ultrathin stent.

 Recent meta-analyses suggest that ultrathin stents yield more favorable results when compared to the latest generation of DESs. Bangalore et al conducted a meta-analysis of randomized trials that compared newer-generation ultrathin strut DES versus thicker strut second-generation DES. Ten trials comprising 11,658 patients were included. Compared to the thicker strut second-generation DES, newer-generation ultrathin strut DES was associated with a 16% reduction in TLF (relative risk (RR), 0.84) driven by lower rates of MI at 1 year. Ultrathin strut DES was also associated with qualitatively lower rates of any ST.^6^ Following this, Madhavan et al executed a random-effects meta-analysis to evaluate ultrathin-strut drug-eluting stents compared to conventional second-generation thin-strut DES. In total, 16 eligible clinical trials encompassing 20,701 patients were analyzed. The average follow-up period was 2.5 years. Ultrathin-strut DES was associated with a 15% decrease in long-term TLF compared with conventional second-generation thin-strut DES [relative risk (RR) 0.85, P = 0.008] driven by a 25% reduction in CD-TLR (RR 0.75, P = 0.005). The reduction of TLF and TVF with ultrathin-strut DES is largely due to a 25% decrease in CD-TLR and a 16% decrease in CD-TVR, both before and after 1 year.^7^

 In this study, Evermine50 showed favorable outcomes compared to other studies using ultrathin stents. Thinner struts cause minimal intrusion into the vessel lumen, leading to maximum in-stent luminal gain. Additionally, they reduce blood flow turbulence, which results in decreased shear stress after implantation. Lower shear stress causes less platelet activation and NIH. Combining these factors leads to fewer acute events, such as early stent thrombosis and in-stent restenosis, over the long term.^9^ The Evermine50 stent’s ultrathin strut design (50 µm) is engineered to optimize flexibility and deliverability without compromising radial strength, effectively mitigating concerns about recoil. This was supported by the 100% procedural success rate and 98% post-procedural TIMI-III flow grade observed in the study, even in cases with long lesions (29.7%) and thrombotic lesions (9.3%). These outcomes demonstrate the stent’s ability to maintain structural integrity and effective expansion across various lesion types. Evermine50 EES showed a very low cumulative rate of MI, MACE, CD-TLR, and ST, although our cohort had a high incidence of diabetes (48.6%), which is an established risk factor for restenosis after PCI.^22^

 There were some limitations to our study. First, this prospective, single-center, single-arm study included a small patient population without a control group for direct comparison. Second, all follow-ups were clinical or telephonic, and no radiological evidence of the outcomes exists. Third, we only included patients with lesion lengths < 44 mm and a reference vessel diameter of 2.00 mm to 4.5 mm. Moreover, long-term follow-up ( > 5 years) is necessary for enhanced robustness of the safety data. This study involved a relatively younger, low-risk population with predominantly shorter and less complex lesions, which may limit the generalizability of the findings to more challenging real-world cases. Finally, while the stent demonstrated excellent performance in this study, its suitability for highly complex lesions, such as tortuous or heavily calcified vessels, remains to be confirmed.

## Conclusion

 This study demonstrated the favorable safety and performance of the ultrathin Evermine50 EES at 36 months. However, further robust evidence through long-term follow-up data or prospective randomized controlled trials is necessary to compare Evermine50 EES to the current second-generation DES.

## Competing Interests

 None to declare.

## Ethical Approval

 The study was performed in accordance with the Declaration of Helsinki and approved by the Institutional Review Board/Institutional Ethics Committee of KLE University (ECR 211/Inst/KA2013).
